# Model Unity and the Unity of Consciousness: Developments in Expected Float Entropy Minimisation

**DOI:** 10.3390/e23111444

**Published:** 2021-10-31

**Authors:** Jonathan W. D. Mason

**Affiliations:** Mathematical Institute, University of Oxford, Oxford OX2 6GG, UK; jonathan.mason@maths.ox.ac.uk

**Keywords:** expected float entropy minimisation, unity of consciousness, model unity, mind-matter models, relational models, typical data, fundamental postulate of EFE minimisation

## Abstract

The unity of consciousness, or, more precisely, phenomenal unity, is an important property of consciousness and an important area of research in mathematical consciousness science and the scientific study of consciousness. Due to the numerous aspects and complexity of consciousness, the property tends to engender loose or inadequate characterizations. In this article, we introduce the concept and mathematical formulation of model unity. A system has model unity if a single relational model, stretched across the whole system, is optimal. Alternatively, model unity may only be present for subsystems, although there may still be unity at some higher level. As a development in the theory of expected float entropy minimisation, such relational models provide an interpretation of system states and the theory may help to provide insights into questions such as why experience of the visual field is unified and why different people do not have a unified consciousness, for example. This article constitutes a relatively small initial study of model unity. Four investigations were undertaken and are given as examples. A postulate is also given, distilling the foundations of EFE minimisation into a clear statement allowing others to consider whether or not the postulate identifies a self-evident fundamental property of consciousness.

## 1. Introduction

This article introduces the concept and mathematical formulation of model unity in the field of mathematical consciousness science (MCS). Importantly, several example applications of model unity are provided and give a demonstration of its agreement with intuition. Model unity is a related and yet different concept to the usual concept of the unity of consciousness. When people talk of the unity of consciousness, or integration, they are usually referring to the somewhat loosely characterized concept called phenomenal unity. In [1], Bayne and Chalmers consider two conscious states as being phenomenally unified “if there is something it is like for the subject to be in both conscious states simultaneously”. In [2], Tononi, Oizumi and Albantakis characterize integration by the following “each experience is (strongly) irreducible to non-interdependent components. Thus, experiencing the word ‘SONO’ written in the middle of a blank page is irreducible to an experience of the word ‘SO’ at the right border of a half-page, plus an experience of the word ‘NO’ on the left border of another half page—the experience is whole. Similarly, seeing a red triangle is irreducible to seeing a triangle but no red color, plus a red patch but no triangle”.

However, in [3], Wiese has pointed out that “if we now try to extrapolate from Tononi’s examples by adding non-visual aspects, we see that the characterizations do not generalize: tactile aspects of my conscious experience are not seamlessly connected to auditory, visual, or cognitive aspects (at least not in the sense that the halves of the visual field are seamlessly connected); and it is easy to imagine a sound without a visual object, or a visual object without a sound. …the way in which the parts of the visual field are experienced together cannot function as a model of how all aspects of my experience are experienced together”. As we will see, model unity is very relevant to these issues and as a mathematical theory has the potential to provide a level of scientific objectivity to exploring the distinctions Wiese raises.

By definition, see more formally below, a given system has model unity if one can do no better than a single relational model stretched across the whole system. Such relational models provide an interpretation of the states of the system. However, for some systems, the model that best fits is not given by one relational model stretched across the whole system but is instead given by the system as a collection of subsystems each having their own individualized and tailored relational model. More complex systems may then have higher levels that span lower subsystems where the lower subsystems together do not have model unity but the higher levels do. Therefore, a given level within a system may not have model unity and in theory the different relational models may relate to different aspects of consciousness. For example, the relational model in visual experience will be a different relational model to that for auditory experience. So model unity is a different and yet connected concept to the normal idea of the unity of consciousness and appeals to Wiese’s observations.

Moreover, due to the objectivity that mathematical methods afford, we should also talk about the disunity of consciousness. We tend not to think of different individuals as being part of the same system but mathematically it’s informative to consider the application of theory to say the “Alice and Bob system” where Alice and Bob are two different people. Initially it might seem ridiculous to suggest we need a theory to show that Alice and Bob have separate conscious experiences. However, what if Alice and Bob’s brains are conjoined perhaps just by one or two synapses or perhaps by millions of connections. How many connections are enough and which connections are the right connections for there to be phenomenal unity involving both brains? Of course the brain itself is in fact two brains as highlighted by the case of Christina Santhouse, see [4], who had a hemispherectomy at age eight but later earned a master’s degree and started a family. Therefore, a normal brain is formed of two interconnected brains, each partly conditioned by the other, which together have phenomenal unity. Consideration of model unity may also be helpful here since a lack of model unity aught to indicate disunity of consciousness unless the unity is coming from some higher system level and model. In such cases the separate models determined can be very different or virtually identical if the lack of overall model unity is only due to the independence of the subsystems involved rather than the subsystems being very different. For example, this article provides strong evidence that for a given layer within Alice and Bob’s visual cortices, when taken together, there would not be model unity. And yet there is also some evidence to suggest that the same layer within Bob’s left and right visual cortices together would have model unity. The example experiments are only preliminary and do not use actual brain data but nevertheless show that model unity gives the right (anticipated) results in these tests. Of course, more experiments and research is needed if model unity is to gain a substantive foothold within MCS.

### 1.1. The Fundamental Postulate of EFE Minimisation

Model unity extends the theory of expected float entropy minimisation (EFE) which first appeared in the literature in 2012; see [5,6,7,8]. EFE minimisation is a mathematical formulation of a largely overlooked and yet arguably fundamental property of consciousness. Put simply, it is the property that consciousness is very smooth, as continuous as possible and free of unnecessary abrupt transitions, pops and crackles etc. In more scientific terms we state the following helpful postulate. It should be noted that the use of the word “interpretation” in the postulate will mean relational model when transferred to the mathematical domain. The relational model creates a context relative to which the system states are meaningful.

**Postulate** **1** (The fundamental postulate of EFE minimisation). *If we suppose that consciousness is given by an interpretation or representation of system states then, notwithstanding the possibility that a system may need to satisfy a number of requirements to be conscious, among the infinitely many possible interpretations, consciousness is given by some form of minimum expected entropy interpretation of system states that yields an experience free of unnecessary discontinuities whilst exhibiting the intrinsic structural regularities of probable system states.*

The postulate mentions “minimum expected entropy interpretation of system states”. The word “expected” is important here. It is not that the interpretation treats each system stare in isolation such that each system state has as few discontinuities as possible. That clearly would not correspond to conscious experience since when we look at a Jackson Pollock painting the geometry of the field of view does not jump to some new geometry relative to which the painting is almost free of abrupt discontinuities. Instead, we mean that the interpretation, i.e. relational model in the mathematical domain, is the same, or at least very stable whilst the system’s bias is stable, for whatever the current system state is. And, given that the system is biased toward being in certain system states over other system states due to a long history of conditioning by sensory inputs, the interpretation is the one for which the more probable system states have minimal abrupt discontinuities while improbable system states are likely to be full of such abrupt artifacts.

**Figure 1:**
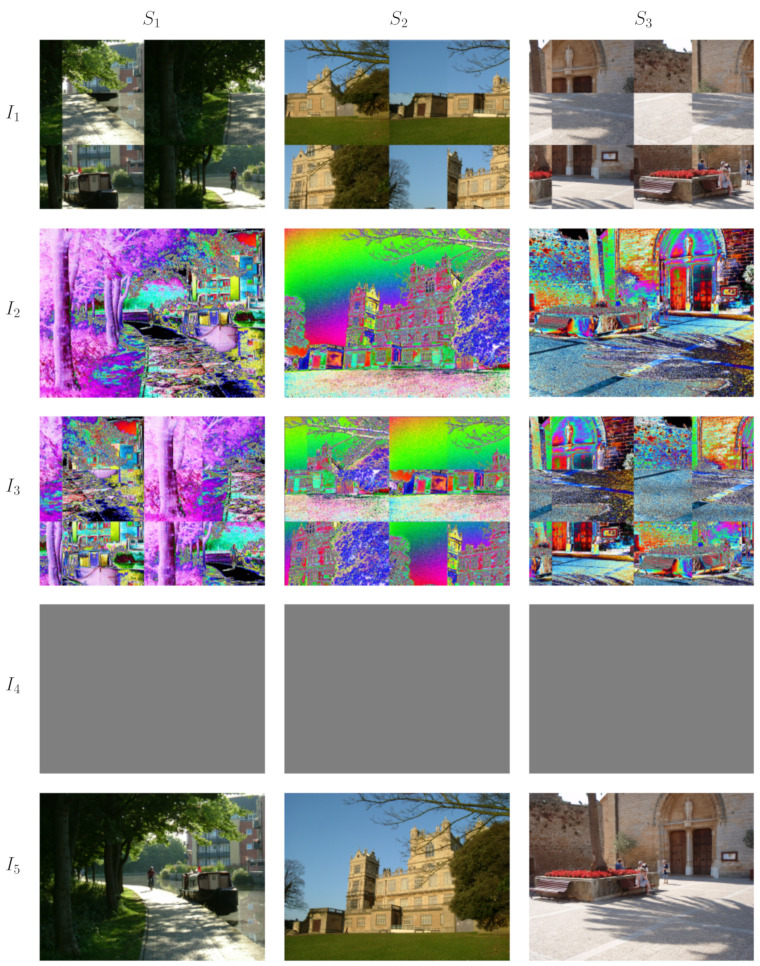
An illustration of the fundamental postulate of EFE minimisation. Three system states are shown, $S_{1},S_{2},S_{3}$ and five examples of interpretations $I_{1}$ to $I_{5}$. $I_{1}$ has unnecessary abrupt discontinuities due to its geometric properties. $I_{2}$ has unnecessary abrupt discontinuities due to the choice of relationships between the colors. $I_{3}$ combines the abrupt discontinuities of both $I_{1}$ and $I_{2}$. $I_{4}$ is nice and continuous but has lost all the details. In this case all of the colors are equally related and there is no relational distinction between them. $I_{5}$ looks to be a good candidate for a minimal expected entropy interpretation of system states.

For those who like the idea of consciousness being given by some form of interpretation of system states there is the explanatory gap of why consciousness is given by one interpretation rather than another. In science it is often the case that a boundary point, limit point or stationary point is important, often referred to as the principle of least action. In the present theory we argue that the interpretation involved is special because it is a limit or boundary point of the set of all possible interpretations of system states since giving the minimum possible expected entropy. Figure 1 provides a visual illustration of the fundamental postulate of EFE minimisation. Note the possibility of auditory analogs of Figure 1 involving abrupt crackles and pops. In Figure 1, while interpretation $I_{4}$ is completely free of discontinuities it is actually a maximum expected entropy interpretation not a minimum. That might come as a surprise to some but to understand why we need to understand something of the notion of entropy being used. An analogy to the common understanding of Boltzmann entropy may help here. Boltzmann entropy is proportional to the log of the number of microstates that satisfy some macroscopic condition. In our case, to calculate the float entropy of a system state $S_{i}$ relative to a particular relational model we first measure the discontinuity (in some suitable sense) of $S_{i}$ given the relationships in the model. That discontinuity measurement is taken as our macroscopic condition and the float entropy of $S_{i}$ is then the log of the number of other system states $S_{j}$ with discontinuity measure less than or equal to that of $S_{i}$ for the given relational model. We see then that a relational model where all of the relationships are identical (that is there are no relational distinctions) will result in the discontinuity measurement being the same for all system states and therefore, every system state will have maximum float entropy. Accordingly the system will have maximum expected float entropy for such a relational model as in the case for $I_{4}$ in Figure 1.

It is hoped that the reader does not find the postulate to be outlandish and that in time consideration may be given to the possibility of the postulate being a point of fact. It is likely that the property of consciousness outlined by the postulate has been largely overlooked because people take the minimal expected entropy aspect of experience for granted, perhaps because we assume that’s how the world outside of our brain is. But, from the author’s experience, a majority of researchers in the field of MCS, and consciousness science in general, think consciousness is most likely to be a property of the brain rather than a property of the brain and the world outside of the brain together. Of course sensory information from the world outside greatly influences brain activity and conditions the brain but we dream with our eyes closed and sometimes of places we have never see. Therefore, if we think that consciousness is a property internal to the brain then clearly the property of consciousness outlined by the postulate is striking and a significant aspect of experience.

Note that consciousness having minimal expected entropy is missing from the list of IIT axioms and postulates (see [2]) even though it is clearly a fundamental aspect of consciousness and, at least on first inspection, does not appear to be implicitly given by the IIT axioms. The postulate is also a useful example of how mathematical consciousness science makes available concepts that aren’t immediately obvious without mathematics.

### 1.2. Expected Float Entropy Minimisation

Before delving into the mathematical details of EFE, it may be helpful to mention a couple of points to kept in mind. Firstly, what follows only involves relational models for the primary relationships that a system may determine. As mentioned earlier in the introduction, many systems will determine a hierarchy of relational models. In particular, secondary models and higher may involve relationships between features such as edges and objects, for example, that are present when system states are interpreted in the context of a primary relational model that a system determines. Accordingly, the mathematical details of EFE below, involving primary relational models, generalise to multi-relational EFE involving higher models. Little work has been undertaken on multi-relational EFE but initial definitions can be found in [5,6]. Secondly, the following talks about the nodes of a system. In the case of the brain, it is important not to immediately assume that a system node is an individual neuron. It is reasonable for a node to be a tuple of neurons in a somewhat analogous way to bytes being the nodes within a synthetic digital representation of sound rather than individual bits. Initial investigations into how systems may determine such a node base are given in [5] and the present article provides a short discussion in Section 4.3.

In the following, we recall the mathematical details of EFE minimisation before giving the mathematical definition of model unity. It will help us with the latter if we slightly simplify the notation used in [5,6] in a similar way to that given in [8].

A system, such as the brain and its subsystems, is assumed to be made up of a finite number of nodes. We denote the set of such system nodes by *S* and take n:=|S|. The range $V:=\{v_{1},v_{2},\ldots ,v_{m}\}$ of states that the nodes can be in is also assumed to be finite. Given *S* and *V*, a system state is equivalent to a mapping,

$$\begin{array}{c} f:S\mapsto V, \end{array}$$

and in this article we denote the set of all such maps by $\Omega_{S,V}$, where $|\Omega_{S,V}|=m^{n}$, and refer to such maps as system states. Informally, the probability of finding the system in system state $f\in \Omega_{S,V}$ is given by a probability distribution $P:\Omega_{S,V}\mapsto \left[0,1\right]$ determined by the system. In practice, *P* is usually unknown and instead a finite set *T* of numbered observations of the system is used. In this case the observations are handled using a function

$$\begin{array}{c} \tau :\left\{1,\ldots ,|T|\right\}\mapsto \Omega_{S,V}, \end{array}$$

so that for the *i*th observation we have τ(i)=f for some $f\in \Omega_{S,V}$. Note that τ need not be injective. In order to move from the above particulars about systems to relational models that such systems may determine we use weighted relations. For any given set *S*, a weighted relation on *S* is a map

$$\begin{array}{c} R:S\times S\mapsto [0,1]. \end{array}$$

We say that *R* is reflexive and symmetric if R(a,a)=1 and R(a,b)=R(b,a), ∀a,b∈S respectively. All of the weighted relations used in this article are reflexive and symmetric and we denote the set of all such relations on a set *S* by $\Psi_{S}$. For notes on the use of reflexive and symmetric relations, see [5,6]. A primary relational model on a system with set of noses *S*, as described above, involves a pair of weighted relations $R\in \Psi_{S}$ and $U\in \Psi_{V}$. Of course, we are looking for a particular pair *R* and *U* that minimise expected float entropy, but to do this requires the following composition. For all $f\in \Omega_{S,V}$ and $U\in \Psi_{V}$ we have the composition

$$\begin{array}{c} U\circ f:=U(f(.),f(.)), \end{array}$$

and note that $U\circ f\in \Psi_{S}$. Then, for all such *f*, *U*, and $R\in \Psi_{S}$, we use the composition to determine the following subset of $\Omega_{S,V}$

(1) $$\begin{array}{c} A\left(R,U,f\right):=\{\tilde{f}\in \Omega_{S,V}:d\left(R,U\circ \tilde{f}\right)\leq d\left(R,U\circ f\right)\}, \end{array}$$

where d is usually taken as the $d_{1}$ metric on the set of all weighted relations on *S*. We can now give the definition of float entropy and expected float entropy as follows. For a system with probability distribution $P:\Omega_{S,V}\mapsto \left[0,1\right]$, let *T* be a finite set of numbered observations of the system, let $R\in \Psi_{S}$ and $U\in \Psi_{V}$. The float entropy of a system state $f\in \Omega_{S,V}$, the expected float entropy of the system, and the approximation of the expected float entropy using *T*, all relative to *R* and *U*, are defined as

(2) $$\begin{array}{c} \mathrm{fe}\left(R,U,f\right):=\log_{2}\left(|A\left(R,U,f\right)|\right), \end{array}$$

(3) $$\begin{array}{c} \mathrm{efe}\left(R,U,P\right):=\sum_{f\in \Omega_{S,V}}P\left(f\right)\mathrm{fe}\left(R,U,f\right), \end{array}$$

(4) $$\begin{array}{c} \mathrm{efe}\left(R,U,T\right):=\frac{1}{\left|T\right|}\sum_{k=1}^{\left|T\right|}\mathrm{fe}\left(R,U,\tau \left(k\right)\right), \end{array}$$

respectively. The primary relational model that a system determines (up to a certain resolution) under EFE minimisation is then

(5) $$\begin{array}{c} R,U=\underset{R\in \Psi_{S},\;U\in \Psi_{V}}{arg min}\mathrm{efe}\left(R,U,P\right). \end{array}$$

In practice we replace efe(·,·,P) in (5) with efe(·,·,T). At this point it may be helpful to add the following note of clarification. We are not saying that a system itself is calculating EFE values and preforming such a minimisation, but instead that, due to the system’s bias, such a minimum solution, when able to be determined, is associated with the system as an emergent property. It is worth noting however that EFE minimisation is a form of machine learning and therefore the system’s bias might be the result of some form of distributed approximate analog to EFE minimisation happening within the system; see [6].

Finally, we should link the obtained primary model in (5) back to the fundamental postulate; Postulate 1. The model provides an interpretation of system states that exhibits the required minimum expected entropy property of consciousness mentioned in the postulate; see [5]. For example, an appropriate application of the theory to the visual system may result in R giving the relationships between points in our perception of the field of view, giving the perceived geometry of the field of view, and U giving the perceived relationships between colours. Of course, as mentioned earlier in the introduction, the primary model is just that “primary” and consciousness is vastly richer requiring many more relational models to be involved in the interpretation of system states. We can now introduce the mathematics behind model unite.

### 1.3. Model Unity

The definition of model unity requires the introduction of two functions μ and *M*. We also require some additional notation. Let *S* be the set of nodes of a system with probability distribution $P:\Omega_{S,V}\mapsto \left[0,1\right]$. We will call X⊆S a subsystem of *S*. We can view *S* as a collection of subsystems and denote the set of all such possible collections by

$$\begin{array}{c} \hat{S}:=\left\{X\subseteq 2^{S}:\cup_{X\in X}X=S,\;\emptyset \notin X,\;S\notin X,\;X\cap Y=\emptyset \;\mathrm{for}\;\mathrm{all}\;X,Y\in X\;\mathrm{with}\;X\neq Y\right\}. \end{array}$$

Mathematically speaking, $\hat{S}$ is the set of all nontrivial partitions of *S* but it is important to avoid confusion here with ideas in IIT. An element $X\in \hat{S}$ is just *S* viewed as a collection of subsystems and no structural changes have been made to the system. We are instead interested in whether it is best to use a single relational model across the whole of a system or whether using a number of individualized models for *S* viewed as a collection of subsystems is better.

Now, the notation introduced in Section 1.2 can be extended. For X⊆S a subsystem of *S*, an element of $\Omega_{X,V}$ is denoted by $f_{X}:X\mapsto V$ and $P_{X}:\Omega_{X,V}\mapsto \left[0,1\right]$ denotes the marginal probability distribution for the subsystem *X* obtained from the distribution $P:\Omega_{S,V}\mapsto \left[0,1\right]$. That is we have

$$\begin{array}{c} P_{X}\left(f_{X}\right):=\sum_{{f|}_{X}=f_{X}}P\left(f\right), \end{array}$$

where the sum is taken over all $f\in \Omega_{S,V}$ and ${f|}_{X}$ is the restriction of *f* to *X*. A point to be aware of is that in theory it is possible to have

$$\begin{array}{c} V_{X}:=\underset{f_{X}\in \mathrm{supp}\left(P_{X}\right)}{⋃}f_{X}\left(X\right)⫋V,\;\mathrm{where}\;\mathrm{supp}\left(P_{X}\right):=\left\{f_{X}\in \Omega_{X,V}:P_{X}\left(f_{X}\right)\neq 0\right\}. \end{array}$$

This would imply that for the subsystem *X* we should perhaps replace the node range *V* with a smaller range $V_{X}$ resulting in a smaller set of possible subsystem states $\Omega_{X,V_{X}}$ when calculating EFE values. However, for systems with comparable nodes, this is a rather contrived situation and usually in practice, as is the case for all subsystems in all of the examples in this article, we do have $V_{X}=V$. This should also be kept in mind when denoting the weighted relations in our primary models for subsystems. In particular, $R_{X}$ denotes an element of $\Psi_{X}$ and $U_{X}$ denotes an element of $\Psi_{V_{X}}$ but in the usual case where $V_{X}=V$ the notation $U_{X}$ is just a way of showing that $U_{X}$ and $R_{X}$ form part of the same primary model.

We can now extend the above notation to definitions (1)–(4) above. We have,

$$\begin{array}{c} A\left(R_{X},U_{X},f_{X}\right):=\left\{\tilde{f_{X}}\in \Omega_{X,V_{X}}:d\left(R_{X},U_{X}\circ \tilde{f_{X}}\right)\leq d\left(R_{X},U_{X}\circ f_{X}\right)\right\}, \end{array}$$

$$\begin{array}{c} \mathrm{fe}\left(R_{X},U_{X},f_{X}\right):=\log_{2}(|A\left(R_{X},U_{X},f_{X}\right)\left|\right), \end{array}$$

$$\begin{array}{c} \mathrm{efe}\left(R_{X},U_{X},P_{X}\right):=\sum_{f_{X}\in \Omega_{X,V}}P_{X}\left(f_{X}\right)\mathrm{fe}\left(R_{X},U_{X},f_{X}\right),\\ \mathrm{efe}\left(R_{X},U_{X},T\right):=\frac{1}{\left|T\right|}\sum_{k=1}^{\left|T\right|}\mathrm{fe}\left(R_{X},U_{X},\tau \left(k\right){|}_{X}\right), \end{array}$$

where ${\tau \left(k\right)|}_{X}$ is the restriction of $\tau \left(k\right)\in \Omega_{S,V}$ to *X*. This is all straightforward and allows us to finally give the definitions of the functions μ and *M*. Let $X\in \hat{S}$, then

(6) $$\begin{array}{c} \mu \left(X,P\right):=\left(\sum_{X\in X}\mathrm{efe}\left(R_{X},U_{X},P_{X}\right)\right)-\mathrm{efe}\left(R,U,P\right), \end{array}$$

where, with reference to (5), each term is individually minimized with respect to the choice of primary models used and the last term is the minimum EFE for the whole system. In practice we use μ(X,T). Furthermore, define

$$\begin{array}{c} M\left(P\right):=\min_{X\in \hat{S}}\mu \left(X,P\right). \end{array}$$

We can now give the definition of model unity.

**Definition** **1** (Model unity). *A system, with probability distribution $P:\Omega_{S,V}\mapsto \left[0,1\right]$ giving the probability of finding the system in any given state, has model unity if and only if M(P)≥0.*

Superficially, it is easy to see why the definition of model unity makes sense. When M(P)≥0 an optimal single model stretched across the whole system gives equal or lower EFE than that given by a number of optimal individualized models when *S* is viewed as any nontrivial collection of subsystems. In other words, we can do no better than to use a single model. However, we should look at *M* and μ more closely.

The intended interpretation of *M* is rather literal and should not be confused with that of ϕ in IIT; see [2]. For a given system with M(P)≥0, we interpret M(P) as quantifying the strength of the system’s model unity, while recalling that the model gives an interpretation of system states. For systems without model unity, M(P)<0, Lemma 1 can be applied. Relevant details are also given in Section 4.2.

**Lemma** **1** (First lemma of model unity). *For a system having a probability distribution $P:\Omega_{S,V}\mapsto \left[0,1\right]$, define*

$$\begin{array}{c} X:=\underset{X\in \hat{S}}{arg min}\mu \left(X,P\right),when\;uniquely\;determined, \end{array}$$

*or let X be any such minimum argument if not unique. Then each subsystem X∈X with |X|>1 has model unity, that is $M(P_{X})\geq 0$. When M(P)<0 and X is unique, every subsystem that is a union of subsystems from the collection X does not have model unity.*

**Proof.**  The proof is almost immediate. Suppose, towards a contradiction, there is subsystem X∈X with |X|>1 and $M(P_{X})<0$. Then, applying the definition of μ to the subsystem, we can replace *X* with some nontrivial partition of *X* giving a new partition $\tilde{X}$ of *S* with $\mu \left(\tilde{X},P\right)<\mu \left(X,P\right)$. This contradicts the definition of X. The second part of the lemma follows in a similar way. □

When a system does not have model unity Lemma 1 and Postulate 1 suggest what to do. At the level of primary models the system *S* should be viewed as the collection of subsystems X defined in Lemma 1. Each subsystem X∈X has its own minimum EFE value and the sum of these individual EFE values (which is the bracketed summation in the definition of μ) gives a collective minimum EFE value for *S* when viewed as the collection of subsystems X. Each subsystem X∈X has its own individualised optimal primary model (when determined) giving an interpretation of the states of the subsystem. Finally, each subsystem X∈X has its own model unity value and has model unity by Lemma 1. The second part of the lemma tells us that we cannot build larger subsystems with model unity out of the subsystems in X. Of course this is still assuming M(P)<0.

Turning our attention to μ, we note that the value of the summation in (6) over the partition X falls within a comparable range to that of the final term which is the minimum EFE for the system as a whole. To show this, we need to express the summation in a different form, which will also reveals another identity for μ. We have,

$$\begin{array}{cc} \sum_{X\in X}\mathrm{efe}\left(R_{X},U_{X},P_{X}\right) & =\sum_{X\in X}\sum_{f_{X}\in \Omega_{X,V}}\left(\sum_{{f|}_{X}=f_{X}}P\left(f\right)\right)\log_{2}(|A\left(R_{X},U_{X},f_{X}\right)\left|\right)\\ \; & =\sum_{X\in X}\sum_{f_{X}\in \Omega_{X,V}}\left(\sum_{{f|}_{X}=f_{X}}P\left(f\right)\log_{2}(|A\left(R_{X},U_{X},f{|}_{X}\right)\left|\right)\right)\\ \; & =\sum_{X\in X}\sum_{f\in \Omega_{S,V}}P\left(f\right)\log_{2}(|A\left(R_{X},U_{X},f{|}_{X}\right)\left|\right)\\ \; & =\sum_{f\in \Omega_{S,V}}\sum_{X\in X}P\left(f\right)\log_{2}(|A\left(R_{X},U_{X},f{|}_{X}\right)\left|\right)\\ \; & =\sum_{f\in \Omega_{S,V}}P\left(f\right)\left(\sum_{X\in X}\log_{2}(|A\left(R_{X},U_{X},f{|}_{X}\right)\left|\right)\right)\\ \; & =\sum_{f\in \Omega_{S,V}}P\left(f\right)\log_{2}\left(\prod_{X\in X}\left|A\left(R_{X},U_{X},f{|}_{X}\right)\right|\right). \end{array}$$

The product in the last equality can be expressed as

$$\begin{array}{cc} \prod_{X\in X}\left|A\left(R_{X},U_{X},f{|}_{X}\right)\right|\; & =\prod_{X\in X}\left|\left\{\tilde{f_{X}}\in \Omega_{X,V_{X}}:d\left(R_{X},U_{X}\circ \tilde{f_{X}}\right)\leq d\left(R_{X},U_{X}\circ f{|}_{X}\right)\right\}\right|\\ \; & \leq \prod_{X\in X}\left|\left\{\tilde{f_{X}}\in \Omega_{X,V}:d\left(R_{X},U_{X}\circ \tilde{f_{X}}\right)\leq d\left(R_{X},U_{X}\circ f{|}_{X}\right)\right\}\right|\\ \; & =\left|\left\{\tilde{f}\in \Omega_{S,V}:\underset{X\in X}{⋀}d\left(R_{X},U_{X}\circ \tilde{f}{|}_{X}\right)\leq d\left(R_{X},U_{X}\circ f{|}_{X}\right)\right\}\right|\\ \; & \leq |\Omega_{S,V}|, \end{array}$$

which, with reference to (1), confirms that the value of the summation in (6) falls within a comparable range to that of the final term in (6). With respect to giving another identity for μ, define

$$\begin{array}{c} A_{X}\left(f\right):=\left\{\begin{array}{cc} \left\{\tilde{f}\in \Omega_{S,V}:\forall X\in X,\tilde{f}{|}_{X}\in A\left(R_{X},U_{X},f{|}_{X}\right)\right\} & \mathrm{if}\exists V_{X}\neq V\\ \left\{\tilde{f}\in \Omega_{S,V}:\forall X\in X,d\left(R_{X},U_{X}\circ \tilde{f}{|}_{X}\right)\leq d\left(R_{X},U_{X}\circ f{|}_{X}\right)\right\} & \mathrm{if}\;\mathrm{all}V_{X}=V. \end{array}\right. \end{array}$$

The identity for μ is then

$$\begin{array}{c} \mu \left(X,P\right)=\sum_{f\in \Omega_{S,V}}P\left(f\right)\log_{2}\left(\frac{|A_{X}\left(f\right)|}{|A_{\left\{S\right\}}\left(f\right)|}\right), \end{array}$$

where $A_{\left\{S\right\}}\left(f\right)$ is just another notation for A(R,U,f), recalling that R,U is the primary model that minimises the EFE of the system when taken as a whole.

Before moving on to the Materials and Methods Section, it may be helpful to consider, all be it very informally, something of why, with regard to primary models, some systems have model unity and others do not. When a system has two or more subsystems that, in some suitable sense, are doing different things or are independent then giving each subsystem its own individualised optimal model will likely be best because (1) the individualised models will fit well whilst a single optimal model stretched across the whole system may fit some subsystems well but possibly not others, and (2) having relational parameters between nodes in the different, possibly independent, subsystems may actually result in a poorer single model. On the other hand, if there is some dependence and some form of regularity of activity across a system then having a single model that stretches across the whole system is likely best because the model benefits from having more parameters; namely the relationship parameters between the subsystems rather than just those within the subsystems. Indeed, very early work on model unity first appeared in Subsection 3.2 of [5] but these initial results involved systems that were too small, |S|=4. In this case we usually have M(P)≥0 because the primary models of nontrivial subsystems have very few parameters in them resulting in very weak models. Having slightly larger systems, |S|=6, makes a big difference and in this case the theory agrees well with intuition, as shown by the results in this article. Future research will be needed to look at still larger systems.

## 2. Materials and Methods

**Figure 2:**
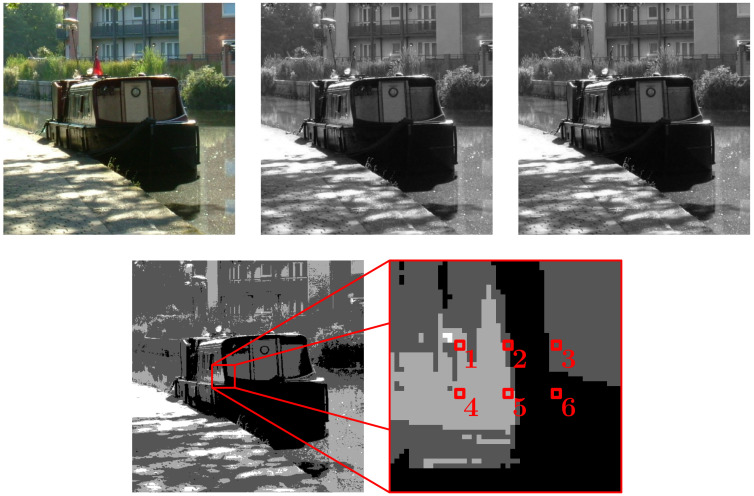
The sampling of a digital photograph using six nodes, |S|=6, and a four shade gray scale, |V|=4. This gives $|\Omega_{S,V}|=4^{6}=4096$. Top-left to bottom-right: the first image is the original, the image is then desaturated and then a contrast enhancement is applied (the contrast enhancement is not needed but may allow the use of a smaller number of samples which is computationally desirable), next the image is posterised (the number of shades is reduced to four giving a four-state node repertoire) and finally the sample is taken. The same sampling method is used for hundreds of images giving hundreds of system states in our sample.

In this section we look at the source of data used in the Results Section examples and also at the optimisation methods used for EFE minimisation. As mentioned in the Introduction, we do not use actual brain data in the examples of this article. A study using actual brain data would need to be undertaken with great care not least because system nodes need not be individual neurons and gathering suitable data would be a challenge. As undertaken in [5,6], in lieu of brain data such as from suitable layers within the visual cortex, we take small samples from hundreds of digital photographs as a simpler and yet somewhat analogous data source. In this case the bias in the data that gives rise to the relationships within the optimal primary models comes directly from the regularities in the world around us rather than from the bias of the brain’s visual system. Figure 2 illustrates the type of sampling method used for the examples in this article. Importantly, for a given investigation, the same method of sampling must be used for all of the digital photographs samples.

The type of sampling method chosen only samples a small part of each image so that the number of independent parameters a primary model has is computationally manageable when undertaking EFE minimisation. As we will see in the Results Section, when sampling data as illustrated in Figure 2, the optimal primary model R, U obtained is such that R recovers the geometry of the sampling grid, which would extend to the whole 2D geometry of the images if all of the pixels were used, and U gives the relationships between the shades of gray recovering the gray scale, and would extend if more colors were used.

Regarding the optimisation method used, because μ is effectively making a comparison between minimum EFE values, it is important to achieve minimum EFE. This has not been a problem in the past but, to be thorougher, in this article two very different optimisation methods were used. Both optimisation methods were applied whenever there was the risk of obtaining a misleading result. The first method used is an iterative grid search; referred to as a binary search in [5,6]. The method uses a multidimensional grid of points in the parameter space. Every grid point is tested using EFE as an objective function and the grid is then re-centered on the best grid point found and scaled down to form a finer and more localised grid. The process is iterated until the difference in minimum EFE values obtained across multiple successive iterations becomes tiny. The assumed minimum EFE and associated primary model are then returned. The method is computationally very expensive and some of the minimum EFE values obtained in this article used over 30 million EFE evaluations. The second optimisation method used is Nelder-Mead; see [9]. This method evaluates the vertices of a general simplex in the parameter space that has one more vertices than the dimension of the space. At each iteration the vertices with greatest valuation is replaced with another point, usually by moving in the direction of the centroid of the remaining vertices. The Nelder-Mead method is an old work horse that has been around since 1965 and is still popular with data scientists today for its simplicity of implementation and efficiency. It is much more efficient than iterative grid search but can also be less reliable. Due to the overlapping use of both optimisation methods the results in the Results Section are robust.

Before moving on to the Results Section it is also worth mentioning that, for the investigations undertaken in this article, significant improvements were made to the efficiency of calculating EFE itself. In particular, before beginning the calculation of float entropy values, the Quicksort algorithm was used to sort the system states $f\in \Omega_{S,V}$ according to the value of d(R,U∘f) shown in (1); and similarly so when calculating EFE for subsystems of *S*. The importance of improving the efficiency of calculating EFE values became clear in undertaking the research for this article because at first a number of potential alternatives to using EFE were tried. In particular, summation ([6] (3)) was found not to behave like EFE unless the probability distribution of the system involved was close to being uniform which is usually not the case. A number of other surrogate functions were also tried but none were able to capture EFE and none worked well enough to be used in place of EFE. These efforts reinforce the use of EFE, as given in the Introduction and going back to [5,6,7], as the correct definition to use in this theory with regards to primary models.

## 3. Results

Exactly four investigations into model unity were undertaken during the research for this article and the results are here presented in four corresponding examples. Each example can simply be taken at face value but in each we also suggest a scenario explaining, in terms of model unity, what the result might mean if it had be obtained for a conscious system.

**Figure 3:**
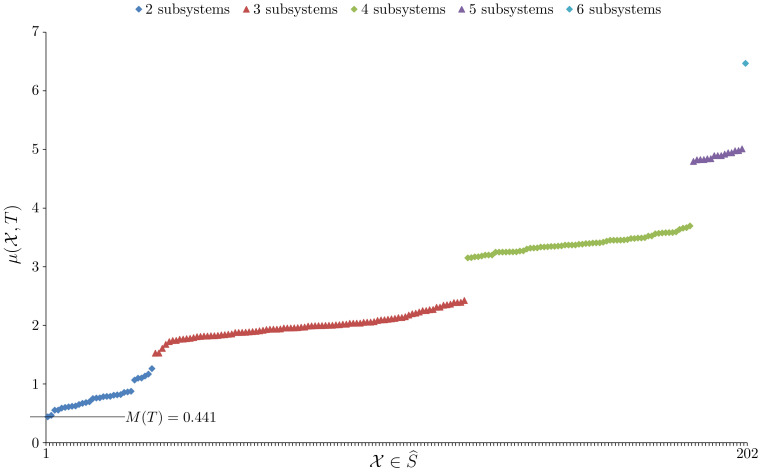
A plot of all μ(X,T) values for Example 1 with the elements of $\hat{S}$ distributed along the *x*-axis and ordered by increasing value of μ.

**Example** **1.** *In this example 410 digital photographs of everyday scenes were sampled, |T|=410, using the method shown in Figure 2. Therefore, |S|=6, |V|=4 and $|\Omega_{S,V}|=4^{6}=4096$. For the system taken as a whole, EFE minimisation gave efe(R,U,T)=5.53207, to six sf. The main question of interest here is whether the system has model unity. Figure 3 plots the μ(X,T) value for every $X\in \hat{S}$, where $\hat{S}$ is the set of all ways of viewing the system S as a nontrivial collection of subsystems. The Bell number $B_{6}=203$, so given $\left\{S\right\}\notin \hat{S}$ we have $|\hat{S}|=202$.*

*From Figure 3 we see that M(T)=0.441 confirming the system has model unity. The primary model, R,U, obtained for the whole system is given in Table 1.*

**Figure 4:**
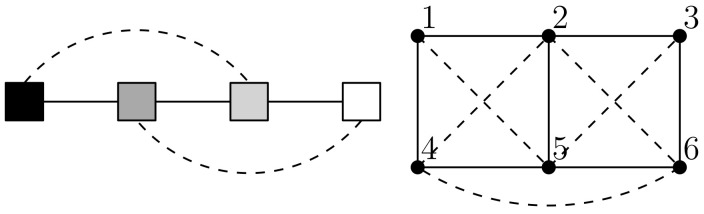
A graph depiction of the primary model in Table 1 showing strongest relationships (*solid lines*) and intermediate relationships (*dash lines*).

*For clarity, Figure 4 depicts the primary model as a graph. For U, relationships of strength above 0.4 are shown by a solid line while intermediate relationships between 0.05 and 0.4 are shown with a dash line. For R, relationships above 0.87 are shown by a solid line while intermediate relationships between 0.74 and 0.87 are shown with a dash line. The primary model corresponds well with what we would anticipate having simultaneously recovered the geometry of the sampling grid and the relationships that give the gray scale. However, there is one intermediate relationship between node 4 and node 6 that is unexpected which we attribute to the sample size being a little too small for the data set used on this occasion.*

**Suggested scenario:** *For a suitable system of nodes of Bob’s visual cortex, Example 1 may suggest that the definitions of this article would yield a result of model unity for such a system. Note that the nodes involved need not be individual neurons and can be tuples of neurons or larger structures. Accordingly, the system as a whole would also yield an associated primary relational model that would provide a minimum expected entropy interpretation of system states.*

**Figure 5:**
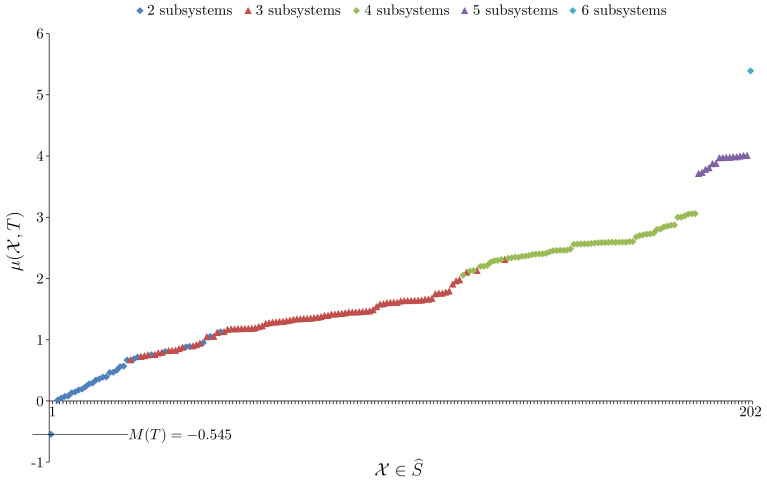
A plot of all μ(X,T) values for Example 2 with the elements of $\hat{S}$ distributed along the *x*-axis and ordered by increasing value of μ.

**Example** **2.** *In this example we are interested in a system with two similarly distributed and yet independent subsystems. To do this we construct the set of system observations T from two sets of observations denoted $T_{\mathrm{Alice}}$ and $T_{\mathrm{Bob}}$. Both use the sampling method shown in Figure 2 except instead of using all six nodes, the $T_{\mathrm{Alice}}$ observations only sample the top row of nodes (nodes 1, 2 and 3) while the $T_{\mathrm{Bob}}$ observations only sample the bottom row of nodes (nodes 4, 5 and 6). The sample sizes are such that $|T_{\mathrm{Alice}}\left|=\right|T_{\mathrm{Bob}}|=200$. The question now arises of how to form T to ensure independence of the subsystems. If we were using probability distributions directly then the answer from information theory is to simply take the joint probability distribution to be the product distribution. In this case the two subsystems would have zero mutual information. When using samples the situation is similar. In this case the Cartesian product $T=T_{\mathrm{Alice}}\times T_{\mathrm{Bob}}$ should be used. This gives a very large sample $\left|T\right|=200^{2}=40,000$ which is why $T_{\mathrm{Alice}}$ and $T_{\mathrm{Bob}}$ are rather small samples. Constructing T in this way ensures that knowing the state of the Alice subsystem tells us nothing about the state of the Bob subsystem beyond what is already known about the Bob distribution and vice versa. The system associated to T has |S|=6, |V|=4 and $|\Omega_{S,V}|=4^{6}=4096$, and, for the system taken as a whole, EFE minimisation gave efe(R,U,T)=6.60773, to six sf. Now, Figure 5 plots the μ(X,T) value for every $X\in \hat{S}$.*

**Figure 6:**
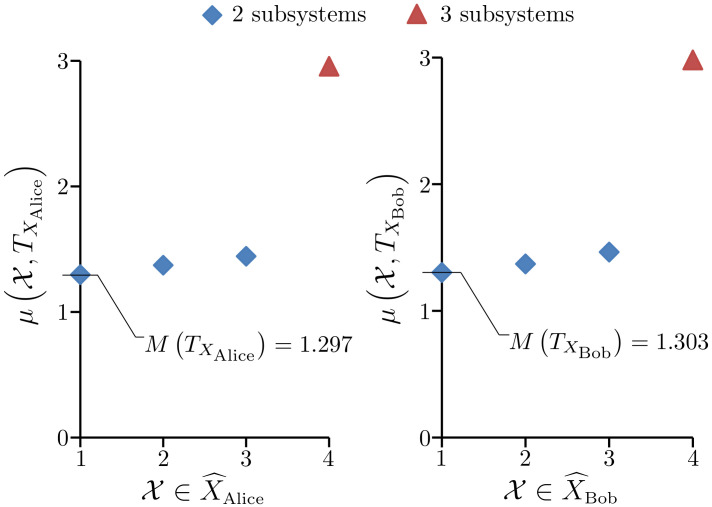
Left: A plot of all $\mu (X,T_{X_{\mathrm{Alice}}})$ values over the elements of ${\hat{X}}_{\mathrm{Alice}}$. Right: A plot of all $\mu (X,T_{X_{\mathrm{Bob}}})$ values over the elements of ${\hat{X}}_{\mathrm{Bob}}$.

*From Figure 5 we see that M(T)=−0.545, so this system does not have model unity. Not surprisingly, the element $X\in \hat{S}$ that gives μ(X,T)=−0.545 is $X=\{X_{\mathrm{Alice}},X_{\mathrm{Bob}}\}$ where $X_{\mathrm{Alice}}$ is the Alice subsystem and $X_{\mathrm{Bob}}$ is the Bob subsystem. By Lemma 1, both subsystems have model unity; the details are given in Figure 6.*

*Therefore, by Lemma 1 and Postulate 1, at the level of primary models we take the system S to be the collection $\left\{X_{\mathrm{Alice}},X_{\mathrm{Bob}}\right\}$ of two separate subsystems. We have $\mathrm{efe}(R_{X_{\mathrm{Alice}}},U_{X_{\mathrm{Alice}}},T)=3.04334$ and $\mathrm{efe}(R_{X_{\mathrm{Bob}}},U_{X_{\mathrm{Bob}}},T)=3.01893$ to six sf, which gives a collective EFE for the two subsystems of 6.06227. The primary models obtained for the subsystems are given in Table 2 and Table 3.*

**Figure 7:**
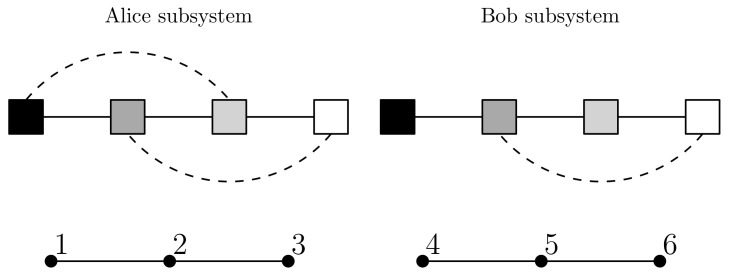
A graph depiction of the primary models in Table 2 and Table 3 showing strongest relationships (*solid lines*) and intermediate relationships (*dash lines*).

*For clarity, Figure 7 depicts the primary models as graphs. For $U_{X_{\mathrm{Alice}}}$ and $U_{X_{\mathrm{Bob}}}$, relationships of strength above 0.2 are shown by a solid line while intermediate relationships between 0.04 and 0.2 are shown with a dash line. For $R_{X_{\mathrm{Alice}}}$ and $R_{X_{\mathrm{Bob}}}$, relationships above 0.84 are shown by a solid line.*

**Suggested scenario:** *For a system with two similarly distributed and yet independent subsystems, where one subsystem is a suitable system of nodes of Alice’s visual cortex and the other subsystem is a suitable system of nodes of Bob’s visual cortex, Example 2 suggest that the definitions of this article would yield a result of model disunity at the level of primary models, that is the system as a whole would not have model unity. Note that the nodes involved need not be individual neurons and can be tuples of neurons or larger structures. Examples 1 and 2 also suggest that the two independent subsystems would each individually as separate systems have model unity. Accordingly, each of the individual subsystems would yield its own associated primary relational model that would provide a minimum expected entropy interpretation of subsystem states for that subsystem. The pair of primary models together would provide a minimum expected entropy interpretation of system states for the system when taken as a whole. Therefore, the two subsystems would have separate individual interpretations according to Postulate 1.*

The next two examples have some similarities with Example 2 and will therefore be presented more expeditiously.

**Example** **3.** *In this example we are interested in a system with two similarly distributed subsystems where the activity in one subsystem lacks regularity with the activity in the other subsystem but the subsystems need not be completely independent. To do this 410 digital photographs of everyday scenes were sampled, |T|=410, using the method shown in Figure 2 except that, for each element of T, nodes 1, 2 and 3 sample a different image to nodes 4, 5 and 6. Therefore, |S|=6, |V|=4 and $|\Omega_{S,V}|=4^{6}=4096$. In this example we denote the two subsystems of interest as X={node1,node2,node3} and Y={node4,node5,node6}. Clearly this example is similar to Example 2 except subsystems X and Y will not be completely independent. To see this, consider a state of X that is rare and only appears once in T; in fact a check of the data revealed there are 16 such states of X that each only appear once in T. Then, given T, knowing X is in the rare state also tells us the state of Y.*

*For the system taken as a whole, EFE minimisation gave efe(R,U,T)=6.73222, to six sf. It was found that M(T)=−0.592, to four sf, so this system does not have model unity. Not surprisingly, the element $X\in \hat{S}$ that gives μ(X,T)=−0.592 is X={X,Y} and, by Lemma 1, both subsystems have model unity; indeed, $M(T_{X})=1.260$ and $M(T_{Y})=1.331$, to four sf. Therefore, by Lemma 1 and Postulate 1, at the level of primary models we take the system S to be the collection {X,Y} of two separate subsystems. We have $\mathrm{efe}(R_{X},U_{X},T)=3.11243$ and $\mathrm{efe}(R_{Y},U_{Y},T)=3.02787$ to six sf, which gives a collective EFE for the two subsystems of 6.14031. The primary models obtained for the subsystems are similar to Table 2 and Table 3.*

**Suggested scenario:** *For a suitable system of nodes of Bob’s brain with two similarly distributed subsystems where the activity in one subsystem lacks regularity with the activity in the other subsystem but the subsystems need not be independent, Example 3 suggests that the definitions of this article would yield a result of model disunity at the level of primary models, that is the system as a whole would not have model unity at the primary model level. Note that the nodes involved need not be individual neurons and can be tuples of neurons or larger structures. Examples 1 and 3 also suggest that the two subsystems would each individually as separate subsystems have model unity. Accordingly, each of the individual subsystems would yield its own associated primary relational model that would provide a minimum expected entropy interpretation of subsystem states for that subsystem. Secondary models and higher may involve relationships between features that are present when the system states are interpreted in the context of the primary relational models and therefore there may be model unity at the level of higher models. Alternatively, if the mode of the system changed (note, it may be that the distribution P changes with mode) such that the activity in one subsystem becomes more regular with the activity in the other subsystem, then the system as a whole may gain model unity at the primary model level. This could have relevance to phenomena such as attention.*

**Example** **4.** *This example is a more extreme version of Example 3. In this example we are interested in a system with two subsystems where not only is the activity in one subsystem lacking regularity with the activity in the other subsystem but the subsystems are also differently distributed. To do this a modification was made to the set of observations used in Example 3. In particular, an arbitrary permutation g:V→V was selected under the conditions that $g\left(v_{i}\right)\neq v_{i}$ for all $v_{i}\in V$ and g is not a relational isomorphism of the gray scale. Then g was applied exactly once to every observation of nodes 4, 5 and 6 in the set of observations used in Example 3. We take this modified set of observations to be our set T of observations in this example and denote the subsystems of interest as X={node1,node2,node3} and $Y_{g}=\left\{\mathrm{node}4,\mathrm{node}5,\mathrm{node}6\right\}$.*

*For the system taken as a whole, EFE minimisation gave efe(R,U,T)=7.31075, to six sf. It was found that M(T)=−1.170, to four sf, so this system does not have model unity. Note that the magnitude of the negative value of M(T) in this example is more extreme than in Example 3. Not surprisingly, the element $X\in \hat{S}$ that gives μ(X,T)=−1.170 is $X=\{X,Y_{g}\}$ and, by Lemma 1, both subsystems have model unity; indeed, $M(T_{X})=1.260$ and $M(T_{Y_{g}})=1.331$, to four sf; which are rightly the same values obtained in Example 3 given the origin of the set T used. Therefore, by Lemma 1 and Postulate 1, at the level of primary models we take the system S to be the collection $\left\{X,Y_{g}\right\}$ of two separate subsystems. We have $\mathrm{efe}(R_{X},U_{X},T)=3.11243$ and $\mathrm{efe}(R_{Y_{g}},U_{Y_{g}},T)=3.02787$ to six sf, which gives a collective EFE for the two subsystems of 6.14031. The primary models obtained for the subsystems are similar to Table 2 and Table 3 with the notable except that the brightness labels in the table for $U_{Y_{g}}$ are permuted according to g. Note that currently unforeseen future refinements to the theory might somehow mitigate the effect on M(T) in this example of applying a simple permutation g to a subsystem to change its distribution. However, in the absence of such a refinement, which is anyway not anticipated, this example supports the following scenario.*

**Suggested scenario:** *For a system with two subsystems, where one subsystem is a suitable system of nodes of Bob’s visual cortex and the other subsystem is a suitable system of nodes of Bob’s auditory cortex, Example 4 suggest that the definitions of this article would yield a result of model disunity at the level of primary models, that is the system as a whole would not have model unity at the primary model level. Note that the nodes involved need not be individual neurons and can be tuples of neurons or larger structures. Examples 1 and 4 also suggest that the two subsystems would each individually have model unity. Accordingly, each of the individual subsystems would yield its own associated primary relational model that would provide a minimum expected entropy interpretation of subsystem states for that subsystem. Secondary models and higher would involve relationships between features that are present when the system states are interpreted in the context of the primary relational models and therefore there may be model unity at the level of higher models. Because the primary model for Bob’s visual system is different to the primary model for Bob’s auditory system, the states of the two systems have different interpretations and so visual experience is different to auditory experience.*

## 4. Discussion

The examples in Section 3 provide the results of four initial investigations into model unity. Although only a small initial study, the results are all as anticipated and provide evidence in support of model unity as a useful concept that may provide a different approach to the unity of consciousness. Example 1 suggests that, for a given sensory cortical system, a suitable system of nodes will have model unity and will therefore determine a single minimum expected entropy interpretation for the states of that system, which corresponds well with the perceived unity of the visual field, for example. Example 2 strongly suggests that independent systems will not have model unity, which corresponds with different people not having a unified consciousness. This leaves open the possibility that two systems may become closer to having model unity due to the presence of mirror neurons. Example 3 suggests that different parts of the brain that are not independent but where there is insufficient regularity in the activity across the systems when taken together will not have model unity at the level of primary models. Model unity may still be present through higher models but this example also raises another interesting possibility. Changes in the brain’s mode and bias (changing *P*) may allow models to unify and separate over time. We discuss this further in Section 4.1. Example 4 suggests that significantly different systems such as the visual cortex and auditory cortex will never have model unity at the level of primary models. While higher models may provide model unity, the different primary models will give different interpretations of system states, which corresponds with visual and auditory experiences being phenomenologically very different.

As mentioned in the Introduction, we don’t assume *M* to be a measure of consciousness. When positive, M(P) quantifies the strength of a system’s model unity. Similarly, when negative, M(P) is quantifying how far from having model unity a system is. As mentioned in [5,6], in this theory, a candidate for a measure of consciousness might involve the shape of EFE as an objective function. That is, if the model that gives the minimum EFE for a system is isolated from other models in the sense that it gives a much lower EFE value than other models do then the system is strongly determining the optimal model. How strongly a system determines its optimal model can be measured from an EFE histogram for the system. In our view, exactly how one would formulate a single measure of consciousness is unclear since consciousness has so many different aspects and therefore likely needs a number of measures to characterize it of which model unity, EFE value, strength of model determination, and model structure, among others, may all have a part to play. However, we do not rule out the possibility of a single measure in some suitable sense.

### 4.1. Model Unity in the Context of Other Theories

As mentioned in the discussion of Example 3 above, changes in the brain’s mode and bias (changing *P*) may allow models to unify and separate over time. If so, then model unity would have significant relevance to theories such as Global Neuronal Workspace (GNW), see [10], that hypothesize the existence of an executive brain system, or global workspace, that selectively gates inputs and outputs mobilizing and suppressing various processing activities. Therefore, as the regularity of activity in different, and yet related, subsystems is modulated, so the model unity of the subsystems when taken together may come and go. Mathematically, there will be useful ways to measure the effect of mode on the model unity of a system. For example, one could try

$$\begin{array}{c} D\left(P,P^{\prime }\right):=\sum_{X\in \hat{S}}\left|\mu \left(X,P\right)-\mu \left(X,P^{\prime }\right)\right|, \end{array}$$

where *P* and $P^{\prime }$ are distributions corresponding to two different system modes of a system. Strong evidence for the existence of at least one type of system modes can be found in [11].

Model unity, as formulated in this article, clearly takes a different approach to Integrated Information Theory (IIT), see [2]. EFE minimisation and model unity prioritize Postulate 1 where as the postulates IIT prioritizes leads the authors to place causation at the very hart of IIT. For a system such as the brain, while causation will clearly be present in how the system’s distribution *P* is determined, EFE minimisation and model unity provide evidence that useful theories can be developed without the need to place causation at the center of their formulation. However, IIT is clearly an important theory since the exact significance of causation in consciousness is still unknown. While our view is that IIT has overlooked Postulate 1 and may have its emphasis in the wrong place, a proper discussion of the issue would require an article of its own. On a constructive note, across the various theories in mathematical consciousness science there is a growing number of quantities such as ϕ, Φ, fe, efe, μ, *M* and mutual information, to name a few, that may all be saying something about consciousness under suitable conditions. Our view, and that of some MCS colleagues, is that a constructive way ahead would be to look for the relationships between such quantities as one might do in physics when determining the relationship between heat and pressure or between current and magnetic field. Such an approach could be very informative providing a better understanding of each of the individual quantities. In such studies one might need to restrict the domain of systems involved to eliminate trivial results. For example, to help prevent trivial differences with IIT results, one might first subject systems to an iterative pruning algorithm where at each iteration, if for all system states a node has no influence on the future of the system or if for all system states it impose no constraints on the system’s past then the node is pruned.

Model unity might have relevance to the Free-energy principal; see [12]. The theory involves a boundary called a Markov Blanket through which a system models the outside world beyond. If a system has model unity and is approximately maximal in the sense that any significant enlargement of the system will not have model unity then in some circumstances the boundary of such a system may have synergies with the concept of a Markov Blanket. It is anticipated that there may be some interesting connections between the theory of the Free-energy principal and the theory of EFE minimisation and model unity. It would also be interesting to compare systems with model unity that are maximal with complexes as defined in IIT, although they will clearly often be different things.

Model unity might find applications in quantum collapse theory. Quantum collapse is an active area of research in mathematical consciousness science due to the possibility that the physical might not be closed; that is consciousness might have some form of direct onward influence on the physical domain, unaccounted for by current physical theories, rather than consciousness just being an epiphenomena (by-product) of the physical; see [8]. The general idea is that some form of protoconsciousness might induce wave function collapse with increasing probability for larger measures of the consciousness-like property. Recent research, see [13], has shown that if the physical is not closed then wave function collapse is the right place to look for such influences. A significant article in this area puts forward the notion of quantum integrated information (QII) inspired by IIT and incorporates a QII dependent term in the evolution equation (Schrödinger equation); see [14]. Regarding model unity, for a given observable and associated operator, the wave function of a system of particles in a superposition can be expanded as a linear sum over the eigenvectors of the operator. The squared modulus of the coefficients gives the probability of the system collapsing to any given eigenstate when observed. This means that the system determines a probability distribution over its set of pure states given by the eigenvectors of the observable. It might therefore be possible to calculate EFE values for the system and find optimal primary models. If so then we can also find the largest subsystem of the particles that has model unity. There are examples of spontaneous collapse models that depend on the mass or number of particles of a system (see [14]) which could be made dependent on the number of particles in the largest subsystem that has model unity. An alternative suggestion that follows different intuition is for the probability of collapse to increase as the value of *M* decreases. Admittedly there is a fair amount of speculation in such suggestion.

### 4.2. Maximal Subsystems with Model Unity

In Section 4.1 we have mentioned the largest subsystem of a system *S* that has model unity. More generally, if a subsystem has model unity but model unity will be lost if it is made any larger then we call the subsystem maximal. We therefore also have the set of all maximal subsystems of a system. If a system has model unity then there will only be one maximal subsystem, namely the system itself. In the case of systems that do not have model unity, Lemma 2 explores how the set of maximal subsystems relates to the set $X:={arg min}_{X\in \hat{S}}\mu \left(X,P\right)$ as given in Lemma 1.

**Lemma** **2** (Second lemma of model unity). *For a system S having a probability distribution $P:\Omega_{S,V}\mapsto \left[0,1\right]$ such that S does not have model unity, define*

$$\begin{array}{c} X:=\underset{X\in \hat{S}}{arg min}\mu \left(X,P\right)\;when\;uniquely\;determined. \end{array}$$

*If the maximal subsystems are all disjoint then X is the set of all maximal subsystems.*

**Proof.**  Let *S* be a system, as described in Lemma 2, such that its maximal subsystems are all disjoint and let *X* be any such maximal subsystem. Suppose toward a contradiction that *X* is not an element of X. We consider the ways in which *X* could fail to be one of the elements of X. Suppose *X* fails to be an element of X by being part of a larger subsystem that is an element of X. By Lemma 1, the elements of X all have model unity but, given *X* is maximal, anything larger would not have model unity; a contradiction. Alternatively, suppose *X* fails to be an element of X by being partitioned in to smaller subsystems that are elements of X. With reference to the definition of model unity and by the uniqueness of X, this conflicts with the definition of X as the minimum argument. Alternatively, suppose *X* fails to be an element of X by being partitioned in to smaller subsystems such that at least one of the smaller subsystems is united with some other subsystem of *S* that is outside of *X*. Let Y⊆X be one such smaller subsystem from the partition of *X* and let Z⊆S\X be the subsystem outside of *X* for which Y∪Z∈X. By Lemma 1, Y∪Z∈X has model unity. Therefore, Y∪Z sits inside some maximal subsystem *W*. Since Z⊆W we have W≠X and yet Y⊆W∩X. This contradicts the maximal subsystems of *S* being disjoint. Therefore, we do have X∈X. Furthermore, every node in *S* sits inside at least one maximal subsystem. Therefore, by the generality of *X*, the only elements of X are maximal subsystems. Finally, the set of all maximal subsystems is unique since it contains every maximal subsystem, and due to all of the maximal subsystems being disjoint the set of all maximal subsystems is a partition of *S*. □

In general maximal subsystems need not be disjoint, although in many such cases pairs of maximal subsystems may come close to being either disjoint or almost identical. When maximal subsystems are not all disjoint there is the potential to obtain misleading results as follows. Since the elements of X are disjoint, if there are maximal subsystems that are not disjoint then there must be at least one maximal subsystem *X* that is not an element of X. If we are not aware of the larger system *S* to which X relates then since *X* has model unity we would just take *X* with its optimal model as our system. However, since *S* has more than one maximal subsystem, *S* does not have model unity and, as outlined in the Introduction following Lemma 1, the right thing to do is to take the system as the collection of subsystems X, which does not include *X*. It may well be that *X* is only slightly different to some particular element of X but there is still a discrepancy because, due to *X* being maximal, *X* can not sit inside some element of X. However, the discrepancy does not indicate any inconsistency in the theory, it simply indicates that a wider context than the current system of interest may be important. It may also be the case that if the maximal subsystems of a system are not all disjoint then each element of X may turn out to be the intersection of maximal subsystems; note that every element of X has model unity and so sits inside one or more maximal subsystems. More research would be needed to determine such a result.

We will conclude the discussion by introducing a potentially useful form of transitivity defined as follows. For a given system *S* having distribution *P*, model unity is transitive if for all subsystems X,Y,Z of *S*, for which subsystems X∪Y and Y∪Z have model unity, the subsystem X∪Y∪Z also has model unity. In general we do not expect model unity to be transitive. However, as we will explain following Corollary 1, model unity is likely to have a bias toward satisfying the transitivity condition, particularly when *Y* is relatively large.

**Corollary** **1.** *For a system S having a probability distribution $P:\Omega_{S,V}\mapsto \left[0,1\right]$ such that S does not have model unity, define*

$$\begin{array}{c} X:=\underset{X\in \hat{S}}{arg min}\mu \left(X,P\right)\;when\;uniquely\;determined. \end{array}$$

*If model unity is transitive on S then X is the set of all maximal subsystems.*

**Proof.**  It is immediate that, if model unity is transitive on *S* then the maximal subsystems are disjoint. The result then follows directly from Lemma 2. □

Now consider any system *S* for which EFE minimisation determines the optimum primary model up to a high level of resolution. Then for subsystems X,Y,Z of *S*, if X∪Y has model unity then it is optimal to have a single model stretched across X∪Y and therefore this single model must be a good fit for *Y*. Similarly if Y∪Z has model unity, the single model stretched across Y∪Z must be a good fit for *Y*. By the near uniqueness of the optimal model on *Y* the model on X∪Y must have a lot in common with the model on Y∪Z. Moreover, if *Y* is large relative to *X* and *Z* then, for a single optimal model stretched across X∪Y∪Z, the model parameters between *X* and *Z* make up a relatively small part of the model. In at least such cases, a single optimal model stretched across X∪Y∪Z may well have lower EFE than the collective EFE given by a number of separate optimal models for any partition of X∪Y∪Z. If so then X∪Y∪Z has model unity. Of course more research is required to determine how usual it actually is for model unity to, at least approximately, satisfy the transitivity condition.

### 4.3. Node Base at the Level of Primary Models

As mentioned in the Introduction, we here include a short discussion concerning the choice of node base at the level of primary models. In the context of the brain it is very tempting to immediately take the nodes of the system to be individual neurons but we know form synthetic digital representations of sound, for example, that the byte locations play the role of nodes not the individual bits locations. It is not clear exactly what the states of an individual neuron are, since states could include firing, not firing, firing frequency, readiness to fire and perhaps more, but it is clear that if we takes a tuple of neurons as a node then the number of states is that of a single neuron to the power of the size of the tuple. Suppose we had a large stack of compact discs (CDs) which all played different music on a standard CD player. Then, intuitively, if we used a different node base to that used for encoding the sound it would likely be difficult to find an alternative choice of CD player, that used the new node base, which would play structured sound for all of the discs. Here by a choice of player we mean a choice of playing order of the nodes and a choice of interpretation of the node states. Therefore, by restricting to highly structured sound the choice of node base is potentially determined. Subsection 3.1 of [5] provides a good introduction to how a system may determine a choice of node base. The initial experiments undertaken show that changing from the sample base to some other base typically has two effects. First, the primary model determined by EFE minimisation is more strongly determined in the sample base. That is if we choose primary models at random and calculate their EFE value then the difference between mean and minimum is greater in the sample base than in the alternative base so that the minimum is more distinct from arbitrary choices of primary model when in the sample base. Secondly, the minimum EFE was less in the sample base than the alternative base in most, but not all, of the experiments. More research is needed with larger systems but the initial results suggest that base may be determined by a system either by the strength that optimal models are determined or directly by EFE minimisation, or by some combination of both.

## 5. Conclusions

In the present article we have introduced the fundamental postulate of EFE minimisation (Postulate 1), introduced the concept and mathematical definition of model unity at the level of primary models as a further development of the theory of EFE minimisation, given the first lemma of model unity (Lemma 1) that identifies subsystems with model unity, undertaken four initial investigations of model unity and alluded to their potential relevance to consciousness through the use of suggested scenarios, considered model unity in the context of other theories and obtained results on maximal subsystems in the form of Lemma 2 and Corollary 1. Node base has also been briefly discussed.

It is hoped that Postulate 1 distills the foundations of EFE minimisation into a clear statement allowing others to contemplate whether or not the postulate has identified a self-evident fundamental property of consciousness. If so then EFE minimisation is a significant theory within mathematical consciousness science. As explained in [5,6], the theory suggests how a system such as the brain may determine the relationships present in consciousness such as those giving the geometry of the field of view, the relationships between colors, the relationships between auditory frequencies and, for higher models, the relationships between objects, sounds and words for example. On reflection, it is clear that consciousness is awash with relationships.

It is important to highlight that the investigations of model unity in this article constitute a relatively small initial study and future research will be needed to establish how well the theory performs for a wider range of systems and for larger systems. Therefore, we leave open the possibility that the theory of model unity may require modification in the future. Not withstanding this is an initial study, all four of the investigations successfully returned the anticipated results according to intuition. The results all supported model unity as a useful concept that is both different and yet related to the usual concept of the unity of consciousness, by which we mean phenomenal unity. In particular, from the suggested scenarios following the investigations, model unity may be able to provide insights into why experience of the visual field is unified, why different people do not have a unified consciousness, how attention might influence the unity of consciousness, and why auditory and visual experiences are phenomenologically different.

We have also indicated the potential relevance of model unity to other theories such as Global Neuronal Workspace theory, the Free-energy principal and quantum collapse theory in mathematical consciousness science. We have also highlighted the need to determine the relationships between the various quantities defined in mathematical consciousness science including those of IIT.

## Figures and Tables

**Table 1:** The primary model obtained in Example 1.

U	**0**	**147.224**	**294.449**	**441.673**		
0	1	0.41970	0.09353	0.00003		
147.224	0.41970	1	0.48171	0.24282		
294.449	0.09353	0.48171	1	0.46463		
441.673	0.00003	0.24282	0.46463	1		
R	**node 1**	**node 2**	**node 3**	**node 4**	**node 5**	**node 6**
node 1	1	0.98568	0.72952	0.98239	0.74209	0.68606
node 2	0.98568	1	0.87753	0.75186	0.93862	0.78366
node 3	0.72952	0.87753	1	0.73635	0.84329	0.99606
node 4	0.98239	0.75186	0.73635	1	0.99130	0.83291
node 5	0.74209	0.93862	0.84329	0.99130	1	0.98446
node 6	0.68606	0.78366	0.99606	0.83291	0.98446	1

**Table 2:** The primary model obtained in Example 2 for the Alice subsystem.

$U_{X_{\mathrm{Alice}}}$	**0**	**147.224**	**294.449**	**441.673**
0	1	0.23437	0.04687	0.01562
147.224	0.23437	1	0.39062	0.07812
294.449	0.04687	0.39062	1	0.45312
441.673	0.01562	0.07812	0.45312	1
$R_{X_{\mathrm{Alice}}}$	**node 1**	**node 2**	**node 3**	
node 1	1	0.98437	0.73437	
node 2	0.98437	1	0.85937	
node 3	0.73437	0.85937	1	

**Table 3:** The primary model obtained in Example 2 for the Bob subsystem.

$U_{X_{\mathrm{Bob}}}$	**0**	**147.224**	**294.449**	**441.673**
0	1	0.21875	0.03125	0.03125
147.224	0.21875	1	0.40625	0.09375
294.449	0.03125	0.40625	1	0.34375
441.673	0.03125	0.09375	0.34375	1
$R_{X_{\mathrm{Bob}}}$	**node 1**	**node 2**	**node 3**	
node 1	1	0.84375	0.71875	
node 2	0.84375	1	0.96875	
node 3	0.71875	0.96875	1	

## Data Availability

Publicly available datasets were analyzed in this study. This data can be found here: https://doi.org/10.5287/bodleian:9ovrBxOw9 (accessed on 31 October 2021).

## Abbreviations

EFE	Expected Float Entropy
GNW	Global Neuronal Workspace
IIT	Integrated Information Theory
MCS	Mathematical Consciousness Science
QII	Quantum Integrated Information

## References

[1] Bayne T., Chalmers D.J., Cleeremans A., Frith C. (2003). What is the unity of consciousness?. The Unity of Consciousness: Binding, Integration, and Dissociation.

[2] Oizumi M., Albantakis L., Tononi G. (2014). From the Phenomenology to the Mechanisms of Consciousness: Integrated Information Theory 3.0. PLoS Comput. Biol..

[3] Wiese W. (2016). How to solve the problem of phenomenal unity: Finding alternatives to the single state conception. Phenom. Cogn. Sci..

[4] Boccella K. (2016). 20 Years after Surgery, a Full Life with Half a Brain.

[5] Mason J.W.D. (2016). Quasi-conscious multivariate systems. Complexity.

[6] Mason J.W.D. (2019). From Learning to Consciousness: An Example Using Expected Float Entropy Minimisation. Entropy.

[7] Mason J.W.D. (2013). Consciousness and the structuring property of typical data. Complexity.

[8] Kleiner J. (2020). Mathematical Models of Consciousness. Entropy.

[9] Nelder J.A., Mead R. (1965). A Simplex Method for Function Minimization. Comput. J..

[10] Dehaene S., Kerszberg M., Changeux J.P. (1998). A neuronal model of a global workspace in effortful cognitive tasks. Proc. Natl. Acad. Sci. USA.

[11] Grindrod P., Lester C. (2021). Cortex-Like Complex Systems: What Occurs Within?. Front. Appl. Math. Stat..

[12] Friston K. (2012). A Free Energy Principle for Biological Systems. Entropy.

[13] Kleiner J., Kremnizer K. Collapse and the Closure of the Physical.

[14] Kremnizer K., Ranchin A. (2015). Integrated Information-Induced Quantum Collapse. Found. Phys..

